# Adding interventions to mass measles vaccinations in India

**DOI:** 10.2471/BLT.15.160044

**Published:** 2016-07-05

**Authors:** Mira Johri, Stéphane Verguet, Shaun K Morris, Jitendar K Sharma, Usha Ram, Cindy Gauvreau, Edward Jones, Prabhat Jha, Mark Jit

**Affiliations:** a Centre de Recherche du Centre Hospitalier de l’Université de Montréal, Tour Saint-Antoine, Porte S03-458, 850 Rue St-Denis, Montréal, Québec, H2X 0A9, Canada.; b Department of Global Health and Population, Harvard TH Chan School of Public Health, Boston, United States of America.; c Division of Infectious Diseases, Department of Pediatrics, University of Toronto, Toronto, Canada.; d National Health Systems Resource Centre, Ministry of Health and Family Welfare, New Delhi, India.; e Centre for Global Health Research, Dalla Lana School of Public Health, Toronto, Canada.; f Department of Infectious Disease Epidemiology, London School of Hygiene & Tropical Medicine, London, England.

## Abstract

**Objective:**

To quantify the impact on mortality of offering a hypothetical set of technically feasible, high-impact interventions for maternal and child survival during India’s 2010–2013 measles supplementary immunization activity.

**Methods:**

We developed Lives Saved Tool models for 12 Indian states participating in the supplementary immunization, based on state- and sex-specific data on mortality from India’s Million Deaths Study and on health services coverage from Indian household surveys. Potential add-on interventions were identified through a literature review and expert consultations. We quantified the number of lives saved for a campaign offering measles vaccine alone versus a campaign offering measles vaccine with six add-on interventions (nutritional screening and complementary feeding for children, vitamin A and zinc supplementation for children, multiple micronutrient and calcium supplementation in pregnancy, and free distribution of insecticide-treated bednets).

**Findings:**

The measles vaccination campaign saved an estimated 19 016 lives of children younger than 5 years. A hypothetical campaign including measles vaccine with add-on interventions was projected to save around 73 900 lives (range: 70 200–79 300), preventing 73 700 child deaths (range: 70 000–79 000) and 300 maternal deaths (range: 200–400). The most effective interventions in the whole package were insecticide-treated bednets, measles vaccine and preventive zinc supplementation. Girls accounted for 66% of expected lives saved (12 712/19 346) for the measles vaccine campaign, and 62% of lives saved (45 721/74 367) for the hypothetical campaign including add-on interventions.

**Conclusion:**

In India, a measles vaccination campaign including feasible, high-impact interventions could substantially increase the number of lives saved and mitigate gender-related inequities in child mortality.

## Résumé

**Objectif:**

Quantifier l'impact sur la mortalité d'un hypothétique ensemble d'interventions à fort impact et techniquement réalisables pour la survie de la mère et de l'enfant lors d'une activité de vaccination antirougeoleuse supplémentaire menée en Inde de 2010 à 2013.

**Méthodes:**

Pour 12 des États indiens participant à l'activité de vaccination supplémentaire, nous avons élaboré des modèles LiST (Lives Saved Tool – Outil de Vies Sauvées) en fonction de données ventilées par État et par sexe sur la mortalité – tirées de l'étude indienne Million Deaths Study –, et sur la couverture des services de santé – tirées d'enquêtes auprès des ménages indiens. Des interventions supplémentaires potentielles ont été identifiées à l'aide d'un examen de la littérature et de consultations d'experts. Nous avons quantifié le nombre de vies sauvées dans le cadre d'une campagne de vaccination antirougeoleuse seule par rapport à une campagne de vaccination antirougeoleuse comportant six interventions supplémentaires (analyse nutritionnelle et alimentation d'appoint pour les enfants, supplémentation en vitamine A et en zinc pour les enfants, supplémentation en micronutriments multiples et en calcium au cours de la grossesse et distribution gratuite de moustiquaires imprégnées d'insecticide).

**Résultats:**

D'après nos estimations, la campagne de vaccination antirougeoleuse a permis de sauver 19 016 enfants âgés de moins de 5 ans. Il a été calculé qu'une hypothétique campagne de vaccination antirougeoleuse incluant des interventions supplémentaires permet de sauver quelque 73 900 vies (étendue: 70 200–79 300), prévenant le décès de 73 700 enfants (étendue: 70 000–79 000) et de 300 mères (étendue: 200–400). Les interventions les plus efficaces se sont avérées être les moustiquaires imprégnées, le vaccin contre la rougeole et la supplémentation préventive en zinc. Les filles représentaient 66% des vies sauvées escomptées (12 712/19 346) dans le cadre de la campagne de vaccination antirougeoleuse et 62% des vies sauvées (45 721/74 367) dans le cadre de la campagne hypothétique incluant des interventions supplémentaires.

**Conclusion:**

En Inde, une campagne de vaccination antirougeoleuse comportant des interventions réalisables à fort impact pourrait considérablement accroître le nombre de vies sauvées et réduire les inégalités entre les sexes concernant la mortalité infantile.

## Resumen

**Objetivo:**

Cuantificar el impacto en la mortalidad del hecho de ofrecer un conjunto hipotético de intervenciones técnicamente viables y de alto impacto para la supervivencia de madres e hijos durante la actividad de inmunización suplementaria antisarampionosa en la India entre 2010 y 2013.

**Métodos:**

Se desarrollaron los modelos de la herramienta “Live Saved Tool” para 12 estados indios participantes en la inmunización suplementaria, en base a información según el estado y el sexo sobre la mortalidad recopilada en el estudio “Million Deaths Study” de la India y sobre la cobertura de servicios sanitarios de las encuestas domésticas realizadas en la India. Se identificaron posibles intervenciones adicionales a través de un análisis de documentos y consultas a expertos. Se cuantificó el número de vidas salvadas durante una campaña que ofrecía la vacuna antisarampionosa frente a una campaña que ofrecía la vacuna antisarampionosa con seis intervenciones adicionales (revisión nutricional y alimentación complementaria para niños, suplementos de vitamina A y zinc para niños, varios micronutrientes, suplemento de calcio durante el embarazo y distribución gratuita de mosquiteros tratados con insecticida).

**Resultados:**

La campaña de vacunación antisarampionosa salvó unas 19 016 vidas de niños menores de 5 años. Se estimó que una campaña hipotética que incluía la vacuna antisarampionosa e intervenciones adicionales salvaría unas 73 900 vidas (alcance: 70 200–79 300), evitando 73 700 muertes infantiles (alcance: 70 000–79 000) y 300 muertes maternas (alcance: 200–400). Las intervenciones más eficaces de todo el paquete fueron los mosquiteros tratados con insecticida, la vacuna antisarampionosa y el suplemento preventivo de zinc. Las niñas representaron un 66% de las vidas salvadas (12 712/19 346) durante la campaña de vacuna antisarampionosa y un 62% de las vidas salvadas (45 721/74 367) durante la campaña hipotética que incluye las intervenciones adicionales.

**Conclusión:**

En la India, una campaña de vacunación antisarampionosa que incluya intervenciones viables y de gran impacto podría incrementar enormemente el número de vidas salvadas y mitigar la desigualdad de género en la mortalidad infantil.

## ملخص

**الغرض:**

قياس مدى التأثير على معدل الوفيات من تقديم مجموعة افتراضية من التدخلات عالية التأثير والممكنة من الناحية الفنية لمعدلات بقاء الأمهات والأطفال على قيد الحياة أثناء فعاليات التحصين التكميلي ضد الحصبة المقامة في الهند في الفترة من عام 2010 إلى عام 2013.

**الطريقة:**

لقد قمنا بإعداد نماذج أداة "Lives Saved Tool" لعدد 12 ولاية هندية مشاركة في فعاليات التحصين التكميلي، استنادًا إلى البيانات الخاصة بمعدل الوفيات بحسب الولاية والنوع من دراسة مليون حالة وفاة في الهند وحول تغطية الخدمات الصحية من المسوحات الأسرية الهندية. تم تحديد بعض التدخلات الإضافية المحتملة من خلال استعراض الكتابات والتشاور مع الخبراء. لقد قمنا بقياس عدد الأرواح التي تم إنقاذها بالنسبة لحملة تطعيمية توفر التطعيم ضد الحصبة فقط مقابل حملة تطعيمية التطعيم ضد الحصبة مع ستة تدخلات إضافية (فحص النظام الغذائي والتغذية التكميلية للأطفال، والمكملات الغذائية من الزنك وفيتامين أ للأطفال، والعديد من المغذيات الدقيقة والمكملات الغذائية من الكالسيوم في مرحلة الحمل والتوزيع المجاني للناموسيات المضادة للحشرات).

**النتائج:**

لقد ساهمت حملة التطعيم ضد الحصبة في إنقاذ حياة ما يقرب من 19016 طفلاً أصغر من 5 أعوام. وكان من المتوقع أن تساهم الحملة التطعيمية الافتراضية التي تشمل التطعيم ضد الحصبة مع التدخلات الإضافية في إنقاذ حياة ما يقرب من 73900 شخصًا (على المدى: 70200 إلى 79300)، ومنع حدوث 73700 حالة وفاة للأطفال (على المدى: 70000 إلى 79000) و300 حالة وفاة للأمهات (على المدى: 200 إلى 400). وكانت أكثر التدخلات فعالية في الحزمة بأكملها هي الناموسيات المضادة للحشرات، والتطعيم ضد الحصبة والمكملات الغذائية من الزنك. وقامت الفتيات بتفسير معدل الأرواح التي تم إنقاذها بنسبة ‏66% (12712/19346) نتيجة للحملة التطعيمية ضد الحصبة، ومعدل الأرواح التي تم إنقاذها بنسبة ‏62% (45721/74367) نتيجة للحملة التطعيمية الافتراضية التي تشمل التدخلات الإضافية.

**الاستنتاج:**

في الهند، يمكن لحملة تطعيمية ضد الحصبة تشمل تدخلات يمكن إجراؤها وذات تأثير مرتفع أن تساهم بشكل كبير في زيادة عدد الأرواح التي يتم إنقاذها والتخفيف من حالات التفاوت المرتبطة بالنوع في وفيات الأطفال.

## 摘要

**目的:**

量化印度 2010-2013 年麻疹强化免疫活动期间,为保障母婴生存提供的一系列技术可行的高影响力假设性干预措施对死亡率的影响。

**方法:**

我们基于《印度百万死亡研究》中各州和各个性别的死亡率以及印度家庭调查的健康服务范围,为参与本次免疫强化活动的 12 个印度邦开发了“挽救生命工具”模型。 通过文献综述和专家咨询确定了可能的附加干预措施。 我们量化了一项仅提供麻疹疫苗的活动和另一项提供麻疹疫苗的同时还配有六项附加干预措施(营养筛查和儿童补充供餐、儿童维生素 A 和锌元素补充、孕期多种微量营养素和钙质补充以及免费发放经杀虫剂处理的蚊帐)的活动各自拯救的生命数。

**结果:**

据估计,该麻疹疫苗活动拯救了 19 016 位 5 岁以下儿童的生命。 一项配有附加干预措施的假设性麻疹疫苗活动预计将拯救大约 73 900 人的生命(范围: 70 200–79 300),使 73 700 位儿童(范围: 70 000–79 000)和 300 位母亲免于死亡(范围: 200–400)。 整个系列中效果最好的干预措施为经杀虫剂处理的蚊帐、麻疹疫苗和预防性锌补充。在麻疹疫苗活动中预计拯救的生命数中,女孩占 66% (12 712/19 346),其中 62% 被拯救生命 (45 721/74 367) 归因于配有附加干预措施的假设性活动。

**结论:**

在印度,配有可行的高影响力干预措施的麻疹疫苗活动可大大增加被挽救生命数,并缓和导致儿童死亡率的性别不平等。

## Резюме

**Цель:**

Оценить, в какой степени предложение гипотетического набора технически исполнимых вмешательств с высоким уровнем воздействия для повышения выживаемости среди матерей и детей могло повлиять на уровни смертности в ходе дополнительного мероприятия по противокоревой иммунизации, проводимого в Индии в 2010–2013 гг.

**Методы:**

Были разработаны модели Инструмента спасенных жизней (Lives Saved Tool) для 12 штатов Индии, участвующих в дополнительной иммунизации, при этом исследователи исходили из конкретных данных о смертности, взятых из исследования «Миллион смертей» (Million Deaths Study) в Индии с разбивкой по штатам и половому признаку, а также из данных об охвате населения Индии службами здравоохранения, полученных путем анкетирования семей. Потенциальные дополнительные вмешательства были определены путем изучения литературных данных и консультирования со специалистами. Количество жизней, которые были спасены в ходе кампании, предлагающей только противокоревую вакцинацию, подсчитали и сравнили с количеством жизней, которые можно было бы спасти в ходе кампании, в которой противокоревая вакцина сочеталась бы с шестью дополнительными вмешательствами (скрининг питания и прикорм детей, введение в детский рацион добавок, содержащих витамин А и цинк, прием беременными множественных добавок, содержащих питательные микроэлементы и кальций, и бесплатное распространение кроватных сеток, обработанных инсектицидами).

**Результаты:**

Вакцинация против кори позволила спасти от смерти примерно 19  016 детей в возрасте младше пяти лет. Гипотетическая кампания, в которой, помимо противокоревой вакцины, применялись бы еще дополнительные вмешательства, согласно прогнозам, могла бы спасти около 73  900 жизней (диапазон полученных оценок от 70 200 до 79  300 человек), из которых в 73  700 случаях была бы предотвращена смерть детей (диапазон значений от 70 000 до 79  000 человек) и в  300 случаях — смерть матерей (диапазон значений от 200 до  400 человек). Из всего набора вмешательств наиболее эффективными оказались бы кроватные сетки, обработанные инсектицидами, противокоревая вакцина и профилактическое употребление цинксодержащих добавок. Из числа всех предположительно спасенных детей девочки составили 66% (12 712 из 19  346 детей) в случае противокоревой вакцинации, а для предполагаемой кампании с дополнительными вмешательствами их доля составила бы 62% (45  721 ребенок из 74 367).

**Вывод:**

В условиях Индии кампания вакцинации против кори, включающая реалистичные и высокоэффективные дополнительные вмешательства, способна значительно увеличить количество спасенных детей и сгладить гендерные различия в показателях детской смертности.

## Introduction

Measles vaccination made an important contribution to the millennium development goal to reduce under-5 mortality (MDG4),^1^ accounting for 23% of the estimated worldwide decline in all-cause child mortality from 1990 to 2008.^2^^,^^3^ A cornerstone of the strategy was that all children be offered a second opportunity to receive a dose of measles-containing vaccine, either through routine immunization services or through mass vaccination campaigns (known as supplementary immunization activities).^4^ Supplemental immunization targets all children, to reach those who have been missed by routine services and also those who may have failed to develop an appropriate immune response after vaccination.^4^ The strategy has been widely implemented in sub-Saharan Africa over the last decade, with measurable success in reducing mortality.^5^ India delayed implementing supplementary immunization, and this may have contributed to the slower decline in measles mortality as compared with sub-Saharan Africa. India’s share of global measles mortality increased from 16% of 535 300 deaths (95% confidence interval, CI: 347 200–976 400) in 2000 to 47% of 139 300 deaths (95% CI: 71 200–447 800) in 2010.^6^

In 2010, India introduced a second opportunity to receive measles-containing vaccine through routine immunization programmes in states with 80% or higher coverage of the first dose of measles-containing vaccine, and elsewhere through supplementary immunization activities. India’s first supplementary mass measles vaccination campaign took place from 2010 to 2013 in 14 states^7^ containing 59% of India’s 113 million under-5 children (authors’ calculations based on census data).^8^ These 14 states have relatively weak health systems compared with the national average^9^ and poorer progress towards MDG4.^10^ The supplementary immunization activity reached 119 million children aged nine months to 10 years, achieving 91% coverage of the target population of 130 743 905.^11^ India’s first round of supplementary mass measles vaccination delivered only a measles-containing vaccine dose. Planning is underway for a larger measles–rubella vaccine introduction campaign targeting children aged 1–15 years.^12^

Campaign-style delivery has two key advantages over routine services; it can achieve high coverage even in areas where the reach of routine services is weak^2^ and it reduces access barriers. On the other hand, a weakness of campaign delivery is that it represents a one-time or cyclic event. Some countries have made strategic use of mass vaccination campaigns to offer additional health interventions such as vitamin A supplements, insecticide-treated bednets and deworming medicines.^2^ Therefore, vaccination campaigns could serve as an important platform to extend the reach of health services to underserved groups and improve maternal and child survival.

To date, India has largely not included add-on interventions with its mass vaccination campaigns and Indian health planners have expressed concerns over the potential challenges of implementing these, while agreeing that add-ons could be beneficial in principle.^9^ To inform the design of future supplementary immunization activities in India and elsewhere we aimed to project the impact on mortality of a hypothetical set of technically feasible, high-impact interventions for maternal and child survival, delivered during India’s 2010–2013 mass measles vaccination campaign.

## Methods

For states participating in the supplementary immunization activity, we conducted a mathematical modelling study to quantify: (i) the number of lives saved by a supplementary immunization activity delivering measles-containing vaccine alone, and (ii) the number of lives that could be saved by a supplementary immunization activity package delivering measles-containing vaccine plus a set of six hypothetical add-on interventions. The analysis baseline reflected existing coverage levels for all interventions offered through routine services. Within each state we also assessed the impact of the interventions on mortality by child’s sex. Ethics approval was not required for this study as it used only secondary data with no personal identifiable information. A technical appendix containing full details of the methods is available from the corresponding author.

### Selection of interventions

We selected add-on interventions for modelling through a literature review and expert consultation. First, we used two systematic reviews to identify maternal and child health interventions that had been linked to routine immunization or vaccination campaigns (but not specific to measles) in a low- or middle-income country, identified from two systematic reviews.^13^^,^^14^ Then we updated the literature search from these reviews to 15 May 2015, and consulted supplementary sources.^2^^,^^15^^–^^17^ Further suggestions were contributed by programme experts, including administrators and managers involved in India’s 2010–2013 measles supplementary immunization activities.^2^^,^^9^ From these inputs we prepared a comprehensive list of potential add-on interventions. Next, we condensed the list based on a review of the evidence of the feasibility of interventions, matched to target population and effectiveness, in the context of a supplementary immunization activity.^3^ Finally, three experts engaged with India’s immunization programme at central and state levels prioritized the interventions to create a shortlist of interventions for analysis based on criteria of programmatic and technical feasibility and policy relevance (Table 1). A total of six interventions – generally offered in India through the routine health system – were selected: (i) nutritional screening of children linked to services for complementary feeding; (ii) vitamin A supplementation for children; (iii) preventive zinc supplementation for children; (iv) free distribution of insecticide-treated bednets; (v) multiple micronutrient supplementation for pregnant women (iron, folic acid, vitamin A); and (vi) calcium supplementation for pregnant women.

**Table 1:** Appraisal of potential add-on interventions for supplementary immunization activities in India

Intervention	Feasible in a single contact	Match to SIA target population	Effective (reduces mortality) in SIA context	Outcome of appraisal
**Child health intervention**				
Nutritional screening	Yes	Yes	Likely	Selected^a^
Vitamin A supplementation	Yes	Yes	Yes	Selected^a^
Promotion of oral rehydration salts or therapy	Yes	Yes	Uncertain	Recommended^b^
Free distribution of oral rehydration salts	Yes	Yes	Uncertain	Recommended^b^
Deworming	Yes	Yes	No	Recommended^b^
Preventive zinc supplementation	Yes	Yes	Uncertain	Selected^a^
Free distribution of insecticide-treated bednets	Yes	Yes	Yes	Selected^a^
Oral polio vaccine	Yes	Yes	No	Recommended^b^
DTP vaccine catch-up/booster dose	Yes	Yes	Likely	Challenging^c^
Japanese encephalitis vaccine	Yes	Yes	Likely	Challenging^c^
Pneumococcal vaccine	Yes	Yes	Likely	Challenging^c^
Rubella (measles–rubella) vaccine	Yes	Yes	Likely	Recommended^b^
Cholera vaccine	Yes	Likely	Yes	Challenging^c^
**Pregnancy intervention^d^**				
Multiple micronutrient supplementation (iron, folic acid, vitamin A)	Yes	To some extent	Yes	Selected^a^
Calcium supplementation	Yes	To some extent	Yes	Selected^a^
Deworming	Yes	To some extent	No	Recommended^b^
Tetanus toxoid vaccine	Yes	To some extent	Yes	Challenging^c^
Promotion of breastfeeding	Yes	To some extent	Uncertain	Potentially valuable^e^
**Additional intervention**				
Family planning	No	Yes	Uncertain	Potentially valuable^e^
Screening for unmet needs and health service referrals	Yes	Yes	Likely	Recommended^b^

SIA: supplementary immunization activity; DTP: diphtheria–tetanus–pertussis.

^a^ Interventions selected for modelling in this analysis.

^b^ Interventions recommended as appropriate but lower priority for this analysis due to low impact on mortality or lack of evidence.

^c^ With the exception of combination vaccines, offering additional vaccines was viewed as challenging due to issues of logistics, safety and human resources.

^d^ Scope for pregnancy interventions depends on the proportion of children brought by mothers to receive measles vaccine and the proportion of pregnant women.

^e^ Interventions judged to be potentially valuable but lower priority for this analysis due to the need for empirical investigation.

Note: Further details of the appraisal are available from the corresponding author.

### Decision modelling

We modelled the impact of the interventions on maternal and child mortality over the period 2009–2013 using the freely available Lives Saved Tool (LiST), version 4.7 (Johns Hopkins Bloomberg School of Public Health, Baltimore, United States of America). LiST is a mathematical model that synthesizes evidence on the causes of maternal and child mortality and the effectiveness of interventions to combat them.^18^ The structure of the model has been described elsewhere.^19^ LiST can be used to project the impact that multiple interventions may have on survival. LiST was chosen because its target populations are similar to those of India’s measles supplementary immunization activities. In addition, validation studies comparing actual measured mortality with modelled mortality showed that LiST provided accurate predictions in diverse geographical settings, including northern India.^20^

### State-level analyses

#### Model parameters

Of the 14 states targeted for supplementary immunization, two were excluded from our analysis, as data on health services coverage (Nagaland, population 1 978 502)^8^ and population structure (Arunachal Pradesh, population 1 383 727)^8^ required for model parameterization were unavailable. LiST developers have made available parameterized models representing India and the state of Bihar in 2008 and we created LiST models for 11 additional supplementary immunization activity states by tailoring the Indian LiST module.

We used recent demographic projections for India to create age- and sex-structured populations for modelling.^10^ Estimates for the effectiveness of add-on interventions were taken from the child health epidemiology reference group (CHERG) systematic reviews incorporated in LiST, with the exception of vitamin A supplementation for which we used a more recent meta-analysis incorporating findings from the Deworming and Enhanced Vitamin A Trial (DEVTA) in Uttar Pradesh, India.^21^ We developed state-specific proportional mortality estimates by mapping cause-of-death data from India’s Million Deaths Study (MDS)^22^^,^^23^ to the LiST model categories. The MDS is a nationally representative longitudinal study of premature mortality monitoring 14 million people in India, which assigns cause of death by physician-reviewed verbal autopsy. For Manipur, Meghalaya and Tripura, state-specific mortality data were not available and for these states we used regional proportional mortality estimates. The MDS did not evaluate pertussis deaths as a separate category due to the difficulty of distinguishing pertussis from causes of death such as pneumonia when using verbal autopsy techniques. We imputed pertussis deaths using CHERG methods.^24^^,^^25^ To characterize immunization coverage before the supplementary immunization activity, values for other parameters were derived from Indian household surveys. The principal data source was India’s 2007–2008 district level household and facility survey;^26^ data were collected just before the measles supplementary immunization. The technical appendix with illustrations of parameter values and data sources for a sample state are available from the corresponding author. Coverage data for the 2010–2013 measles supplementary immunization were provided by the Government of India.

#### Integrated vaccination campaign package

We modelled the supplementary immunization activity as occurring in all states in a single year (2010). The campaign would confer a one-time increase in measles vaccination coverage. Some of the hypothetical interventions, such as delivering vitamin A supplements and carrying out nutritional screening, could be completed at the time of vaccination. For these interventions, increases in coverage were modelled as a function of measles-containing vaccine coverage achieved by the supplementary immunization. Vitamin A supplements for children should be given twice per year; a single dose of vitamin A represents half the annual recommended dose. We therefore calculated the increase in vitamin A coverage conferred by the supplementary immunization as: achieved coverage divided by 2. Nutritional screening is effective only when linked to programmes and services. Based on expert opinion, we assumed that 90% of children screened and found nutritionally deficient would be linked to follow-up services, including complementary feeding, through programmes such as India’s integrated child development services. Several other interventions would require additional follow-through to be effective. For three interventions (multiple micronutrient and calcium supplements for pregnant women and preventive zinc for children) we modelled the causal chain between being offered in the supplementary immunization activity and increased intervention coverage as depending on measles-containing vaccine achievement and compliance. For these interventions, we used an average compliance scenario of 70% and considered two additional scenarios bounding reasonable ranges of low (50%) and high (90%) compliance. We assumed that 73% of freely distributed long-lasting insecticide-treated bednets would be used.^27^ The analytic assumptions are outlined in Table 2 (available at: http://www.who.int/bulletin/volumes/94/10/15-160044), with further details available from the corresponding author.

**Table 2:** Assumptions used in the analysis of measles vaccine with a package of six add-on interventions for the supplementary immunization activity in India

Assumption	Value	Source
**Efficacy of measles vaccine in reducing measles mortality**	0.85	Published study^18^
**Duration of benefit conferred by SIA interventions**		
Insecticide-treated bednets	3 years	Published study^28^
Measles vaccine	Lifelong (beyond analysis timeframe)	Published study^29^
All other interventions^a^	< 1 year	Published study^18^
**Proportion of individuals likely to use SIA add-on interventions**		
Insecticide-treated bednets	73%	Published study^27^
Multiple micronutrient supplements for pregnant women	70% compliance (50% low; 90% high)^b^	Expert opinion and field data
Calcium supplements for pregnant women	70% compliance (50% low; 90% high)^b^	Expert opinion and field data
Preventive zinc for children	70% compliance (50% low; 90% high)^b^	Expert opinion
**Proportion of children identified in the SIA as having nutritional deficiencies and linked to services for complementary feeding and supplementation**	90%^c^	Expert opinion
**Proportion of pregnant women reached by the SIA**	Varies by state, range 21% to 52%	Calculated
**Increase in vitamin A coverage achieved through the SIA**	SIA coverage divided by 2^d^	Calculated
**SIA reaches male and female children equally**	–	Published study^30^
**SIA does not reduce coverage of routine services**	–	Assumed

SIA: supplementary immunization activity.

^a^ This category comprises nutritional screening; vitamin A supplementation for children; preventive zinc supplementation for children; multiple micronutrient supplementation in pregnant women (iron, folic acid, vitamin A); and calcium supplementation in pregnant women.

^b^ Further details of compliance scenarios are available from the corresponding author.

^c^ This estimate for linkage to services is based on the mandate and capacity of India’s integrated child development services programme.

^d^ As Vitamin A supplements should be given twice per year, this represents half an annual dose.

#### Sensitivity analyses

Additional analyses explored the effect of using different sources of data for proportional mortality (i.e. comparing proportional mortality data for India from CHERG and state-specific proportional mortality data from the MDS). We also quantified the impact of parameter uncertainty related to the effectiveness of vitamin A supplementation on diarrhoea mortality for children aged 6–59 months. To do this we contrasted the DEVTA meta-analysis midpoint estimate of 11%^21^ with the 47% mortality benefit incorporated in LiST.^18^ Finally, we developed the Dynamic Measles Immunization Calculation Engine, a transmission dynamic measles model^31^ that enabled us to consider factors not captured in LiST, such as age-specific vaccine efficacy for measles first and second doses, and herd immunity, to model the impact on mortality of the supplementary immunization activity delivering only measles-containing vaccine.

#### Equity impact

Equity analyses were done on a state-by-state basis and assumed that increases in supplementary immunization coverage reached both sexes equally. To quantify the impact of the mass vaccination campaign on sex differences in child mortality, we used sex-specific proportional mortality data from the MDS^22^ and sex-specific coverage data from the third round of the district level household and facility survey^26^ and other sources (further details are available from the corresponding author). We assessed the impact on gender equity of the campaign delivering measles vaccine only and the hypothetical campaign delivering measles vaccine and add-on interventions by comparing the proportion of hypothetical lives saved by the campaign for girls versus boys, and the under-5 mortality rate per 1000 live births for girls versus boys in the years before (2009) and after (2010) the measles campaign.

## Results

### Overall results

India’s decision to introduce a second opportunity for measles vaccination via mass vaccination campaign saved the lives of an estimated 19 016 under-5 children in the 12 states included in our analysis, of whom 11 121 (58%) were in the state of Uttar Pradesh (Table 3).

**Table 3:** Estimated number of under-5 lives saved in 12 states participating in India’s 2010–2013 measles supplementary immunization activity

State	No. (%)	
	Under-5 lives saved by measles vaccine	Under-5 population in SIA states^a^
Assam	378 (2.0)	3 556 222 (4.8)
Bihar	3 436 (18.1)	13 811 150 (19.2)
Chhattisgarh	79 (0.4)	3 014 655 (3.8)
Gujarat	262 (1.4)	6 293 984 (8.2)
Haryana	435 (2.3)	2 763 215 (3.6)
Jharkhand	353 (1.9)	4 022 926 (5.5)
Madhya Pradesh	1 864 (9.8)	8 899 016 (11.2)
Manipur	32 (0.2)	257 601 (0.4)
Meghalaya	33 (0.2)	416 638 (0.6)
Rajasthan	996 (5.2)	8 852 191 (11.0)
Tripura	27 (0.1)	339 014 (0.5)
Uttar Pradesh	11 121 (58.5)	24 945 895 (30.7)
All states^b^	19 016 (100.0)	77 172 507 (100.0)^c^

SIA: supplementary immunization activity.

^a^ Author’s calculations based on Government of India census statistics.^8^

^b^ Including all participating states in the 2010–2013 measles SIA.

^c^ SIA states contained 59% of the under-5 population of India.^8^

Note: Analyses use proportional mortality from the Million Deaths Study^22^ and vitamin A effectiveness from the Deworming and Enhanced Vitamin A Trial.^21^ Lives saved were calculated for the period 2010–2013.

Table 4 shows the projected lives saved in these states by a hypothetical supplementary immunization package that included measles vaccine and high-impact add-on interventions for children and pregnant women. This was based on a scenario of 70% compliance with interventions (when applicable) and on mortality data from India’s MDS. Maternal lives saved were due to calcium supplementation in pregnancy; all other lives saved represented under-5 children. Summing over all states, including maternal and child health interventions in the measles supplementary immunization campaign would have saved around 73 900 (range: 70 200–79 300), preventing 73 700 child deaths (range: 70 000–79 900) and 300 maternal deaths (range: 200–400). The hypothetical campaign offering measles vaccine with add-on interventions was therefore projected to increase the number of lives saved by a factor of 3.89 (range: 3.69 to 4.17) compared with offering measles vaccine alone. The benefits of the add-ons were also distributed among states more closely to the proportion of the population (Table 3 and Table 4; supplementary data are available from the corresponding author). For example, Uttar Pradesh, which had 30.7% of the under-5 target population for the supplementary immunization, gained 58.5% of lives saved from the measles-only supplementary immunization (Table 3) and 36.0% of lives saved from the package of measles supplementary immunization with add-ons (Table 4).

**Table 4:** Projected number of lives saved due to a hypothetical package of measles vaccine with a set of additional maternal and child health interventions during the measles supplementary immunization activity, India 2010–2013

Compliance scenario and state		No. of lives saved											
	Due to specific intervention^a^									Not attributable to specific intervention			Total
	Measles vaccine		Bednets	Zinc	Complementary feeding	Micronutrients	Vitamin A	Calcium^b^		Diarrhoea	Pneumonia	Measles	
**By state (70% scenario)**													
Assam	365		3 611	810	254	149	131	16		121	93	31	5 581
Bihar	3 265		3 981	3 030	1 076	595	415	43		417	421	169	13 412
Chhattisgarh	76		1 691	301	123	126	41	10		33	56	8	2 465
Gujarat	256		733	677	156	250	107	11		63	75	53	2 381
Haryana	421		374	246	61	122	50	5		23	19	15	1 336
Jharkhand	338		2 945	773	192	172	101	13		92	100	24	4 750
Madhya Pradesh	1 791		4 240	2 163	532	499	264	28		234	301	97	10 149
Manipur	31		177	62	42	12	8	1		46	53	35	467
Meghalaya	32		180	82	9	14	10	1		48	56	35	467
Rajasthan	962		2 256	1 604	268	479	175	34		95	204	41	6 118
Tripura	27		206	69	9	10	10	1		4	6	1	343
Uttar Pradesh	10 671		4 539	5 712	1 562	1 333	781	108		662	643	449	26 460
**All states**													
70% scenario	18 235		24 933	15 529	4 284	3 761	2 093	271		1 838	2 027	958	73 929
50% scenario	18 314		24 929	13 346	4 292	2 687	2 109	196		1 647	1 806	829	70 155
90% scenario	18 159		24 934	19 849	4 235	4 838	2 080	350		1 870	2 061	945	79 321

^a^ Lives saved due to the intervention listed in the column header.

^b^ These are maternal deaths; all other deaths represent children younger than 5 years.

^c^ Additional lives saved due to prevention of disease (not intervention-specific).

Note: Including 12 states participating in India’s 2010–2013 measles supplementary immunization activity. Analyses use proportional mortality from the Million Deaths Study^22^ and vitamin A effectiveness from the Deworming and Enhanced Vitamin A Trial.^21^ Lives saved were calculated for the period 2010–2013. Compliance figures apply to multiple micronutrients and calcium for pregnant women, and preventive zinc for children; compliance scenarios are outlined in Table 2.

We explored which add-on interventions in the whole package contributed most to the anticipated reductions in mortality (Table 4). Summing over all states for the average (70%) compliance scenario, the effectiveness of the interventions in descending order were: insecticide-treated bednets (24 933 lives saved), measles vaccine (18 235), preventive zinc supplementation (15 529), complementary feeding (4284), vitamin A supplementation (2093), multiple micronutrients supplementation during pregnancy (3761) and calcium supplementation during pregnancy (271). Some of the lives saved by the supplementary immunization with add-ons could not be attributed to specific interventions and are presented instead by syndrome, including diarrhoea (1838 lives saved), pneumonia (2027) and measles (958). Applying the low (50%) compliance scenario over all states resulted in the same ranking of interventions, while for the high (90%) compliance scenario, the interventions in descending order of importance were: bednets, zinc, measles vaccine, multiple micronutrients, complementary feeding, vitamin A and calcium. 

The contribution to mortality reduction of specific interventions varied considerably among states, reflecting differences in local epidemiology and coverage of health services before the measles vaccination campaign. For the average compliance scenario, measles vaccine conferred 3% of the anticipated benefit in Chhattisgarh (i.e. 76/2465 lives saved) and 40% in Uttar Pradesh (10 671/26 460); insecticide-treated bednets conferred 17% of the benefit in Uttar Pradesh (4539/26 460) and 69% in Chhattisgarh (1691/2465); while zinc conferred 12% of the benefit in Chhattisgarh (301/2465) and 28% (677/2381) in Gujarat (Table 4).

### Sensitivity analyses

Using different assumptions about proportional mortality affected the findings shown in Table 4. Proportional mortality based on the MDS data attributed greater importance to malaria deaths and hence a higher projected number of lives saved by insecticide-treated bednets (24 933/73 929 lives saved across all states) than did proportional mortality based on the CHERG data (4591/69 912 lives saved). Uncertainty concerning the effectiveness of vitamin A supplementation in reducing diarrhoea deaths among children aged 6–59 months had an important impact; higher CHERG values appreciably increased the projected lives saved due to administration of vitamin A (6637/77 979) compared with the DEVTA trial values (2093/73 929). A dynamic model replicating India’s 2010–2013 measles supplementary immunization activity projected that a one-time supplementary immunization delivering only measles-containing vaccine would have saved 47 625 to 95 249 lives of under-5 children, assuming a case-fatality ratio of 1–2%.^6^^,^^32^

### Equity analyses

The campaign mitigated pre-existing inequalities in mortality between girls and boys. For under-5 children, the supplementary immunization delivering only measles-containing vaccine were projected to save the lives of 12 712 (66%) girls and 6 635 (34%) boys. The hypothetical campaign delivering measles vaccine and add-on interventions, based on the 70% compliance scenario, saved an expected 45 721 (62%) girls and 28 647 (39%) boys (Table 5; additional supplementary data are available from the corresponding author). For the eight states in which under-5 mortality per 1000 live births was initially greater in girls than in boys, the gender disparity was reduced by the supplementary immunization activity offering measles-containing vaccine only and further reduced by the hypothetical campaign delivering measles vaccine and add-on interventions (Table 6).

**Table 5:** Projected number of lives saved for under-5 children due to measles vaccine only or due to a hypothetical package of measles vaccine with a set of additional maternal and child health interventions during the measles supplementary immunization activity, India 2010–2013, by child’s sex

State	Lives saved by measles vaccine only				Lives saved by measles vaccine with add-on interventions^a^		
	Total, no.	Girls, no. (%)	Boys, no. (%)		Total, no.	Girls, no. (%)	Boys, no. (%)
Assam	379	242 (64)	138 (36)		5 579	3 090 (55)	2489 (45)
Bihar	3 439	2 231 (65)	1 209 (35)		13 373	8 456 (63)	4 917 (37)
Chhattisgarh	77	34 (45)	42 (55)		2 438	1 169 (48)	1 270 (52)
Gujarat	299	200 (67)	99 (33)		2 396	1 397 (58)	999 (42)
Haryana	430	269 (62)	161 (38)		1 334	850 (64)	484 (36)
Jharkhand	355	225 (63)	130 (37)		4 695	2 493 (53)	2 202 (47)
Madhya Pradesh	1 899	999 (53)	900 (47)		10 379	5 730 (55)	4 649 (45)
Manipur	32	14 (45)	18 (55)		329	158 (48)	171 (52)
Meghalaya	33	15 (46)	18 (54)		337	164 (49)	173 (51)
Rajasthan	1 080	831 (77)	249 (23)		6 292	4 268 (68)	2 024 (32)
Tripura	27	13 (49)	14 (51)		376	199 (53)	176 (47)
Uttar Pradesh	11 296	7 639 (68)	3 657 (32)		26 839	17 747 (66)	9 093 (34)
**Total^b^**	**19 346**	**12 712 (66)**	**6 635 (34)**		**74 367**	**45 721 (61)**	**28 647 (39)**

^a^ Add-on interventions were nutritional screening linked to complementary feeding; vitamin A supplementation for children; preventive zinc supplementation for children; free distribution of insecticide-treated bednets; multiple micronutrient supplementation in pregnant women (iron, folic acid, vitamin A); and calcium supplementation in pregnant women.

^b^ State totals represent the sum of sex-specific models and thus differ from Table 3.

Note: Including 12 states participating in India’s 2010–2013 measles supplementary immunization activity. Analyses use proportional mortality from the Million Deaths study^22^ and vitamin A effectiveness from the Deworming and Enhanced Vitamin A Trial.^21^ Lives saved were calculated for the period 2010–2013. Data are based on the 70% compliance scenario (see Table 2) and apply to multiple micronutrients and calcium during pregnancy, and preventive zinc for children.

**Table 6:** Projected under-5 mortality in the years before (2009) and after (2010) the measles supplementary immunization activity, India 2010–2013, by child’s sex

State						Measles vaccine only											Measles vaccine with add-on interventions^a^				
			Deaths per 1000 live births						Difference (2010–2009)^b^				Deaths per 1000 live births							Difference (2010–2009)^b^	
	Girls				Boys				Girls	Boys	Girls					Boys				Girls	Boys
	2009	2010			2009		2010				2009			2010		2009		2010			
Assam	87.0	86.4			79.0		78.7		−0.6	−0.3	87.9			83.4		79.9		76.3		−4.5	−3.6
Bihar	68.0	66.3			60.0		59.0		−1.7	−1.0	68.3			63.3		60.3		57.4		−5.0	−2.9
Chhattisgarh	61.0	60.9			61.0		60.9		−0.1	−0.1	62.2			59.8		62.2		59.9		−2.4	−2.3
Gujarat	60.0	59.7			52.0		51.9		−0.3	−0.1	60.5			58.9		52.5		51.4		−1.6	−1.1
Haryana	59.0	58.0			50.5		51.0		−1.0	0.5	60.2			57.5		52.2		50.7		−2.7	−1.5
Jharkhand	59.0	58.2			59.0		58.7		−0.8	−0.3	59.9			56.1		59.9		56.7		−3.8	−3.2
Madhya Pradesh	85.0	83.9			82.0		81.0		−1.1	−1.0	85.4			81.0		82.4		78.9		−4.4	−3.5
Manipur	79.0	78.5			79.0		78.3		−0.5	−0.7	89.5			87.7		89.5		87.5		−1.8	−2.0
Meghalaya	79.0	78.6			79.0		78.5		−0.4	−0.5	89.8			88.0		89.8		87.8		−1.9	−2.0
Rajasthan	79.0	78.0			60.0		59.7		−1.0	−0.3	79.5			75.7		60.5		58.7		−3.8	−1.8
Tripura	79.0	78.6			79.0		78.6		−0.4	−0.4	87.2			84.6		87.2		85.0		−2.6	−2.2
Uttar Pradesh	87.0	83.8			71.0		69.5		−3.2	−1.5	87.2			81.1		71.2		68.1		−6.1	−3.1

^a^ Add-on interventions were nutritional screening linked to complementary feeding; vitamin A supplementation for children; preventive zinc supplementation for children; free distribution of insecticide-treated bednets; multiple micronutrient supplementation in pregnant women (iron, folic acid, vitamin A); and calcium supplementation in pregnant women.

^b^ Results were calculated without applying background trends in acquired immune deficiency syndrome mortality.

Note: Including 12 states participating in India’s 2010–2013 measles supplementary immunization activity. Analyses use proportional mortality from the Million Deaths Study^22^ and vitamin A effectiveness from the Deworming and Enhanced Vitamin A Trial.^21^ Lives saved were calculated for the period 2010–2013.

## Discussion

Measles vaccination is important for reduction of child mortality, yet global coverage of the first dose of measles-containing vaccine has been stagnant since 2009.^1^ Mass vaccination campaigns are resource-intensive and planners must assess their value among a range of options for health improvement and resource expenditure. Our analysis demonstrated that India’s introduction of a second opportunity for measles vaccination through large-scale campaigns from 2010 to 2013 made an important contribution to reducing mortality from measles. Our model-based analysis of 12 of the 14 participating states found that India’s measles supplementary immunization activity likely saved the lives of approximately 19 000 under-5 children, corresponding to roughly 29% (range: 24% to 35%) of India’s annual measles mortality.^6^ We also found that a hypothetical supplementary immunization package delivering measles vaccine and a set of additional interventions of known effectiveness would increase the impact on mortality of the mass measles vaccination campaign more than threefold. Despite variation among states, the most important interventions in the package overall were insecticide-treated bednets, measles vaccine and preventive zinc supplementation. This reflects the high burden of infectious disease and undernutrition among Indian children, the impact of malaria in some areas and the relatively low coverage of these key interventions.^24^^–^^26^

Child mortality in India differs markedly by sex, with higher mortality rates recorded for girls.^10^^,^^22^ Caregiver bias associated with preference for a male child likely contributes to the mortality differentials due to lower use of regular health services for girls. Vaccination campaigns, however, show a more equal pattern of use.^30^ We found that, due to the high coverage achieved in states with weak health systems, supplementary immunization helped to mitigate gender-related inequities in child mortality. We also studied differences in mortality for subgroups defined by household wealth status (quintiles) and area of residence (rural/urban). However, the results were uninformative due to the absence of state- and stratum-specific proportional mortality data (available from the corresponding author).

The components of this integrated health package for a supplemental mass vaccination campaign were designed based on a systematic appraisal of the evidence and a quantitative projection of likely impact. Add-on interventions were systematically selected using the published scientific literature and expert guidance.^9^ A key advantage of this approach is that it presents the evidence and assumptions in a transparent framework that permits alternatives to be explored. We placed particular emphasis on defining options for analysis that were evidence-based, feasible and relevant to the Indian context. Cause-of-death data from India’s MDS^22^ enabled us to show state- and sex-specific mortality patterns, while data from recent household surveys^26^ facilitated an accurate portrayal of health services coverage. LiST is a validated policy model that enables competing mortality risks to be considered.^19^ In addition, we validated the LiST projections of the impact of measles-containing vaccine using a transmission dynamic model that takes into account both herd immunity and age-specific vaccine efficacy. As anticipated, the dynamic model results were consistent with LiST results but showed a somewhat higher impact on mortality for the supplementary immunization with measles vaccine.

We highlight five limitations of the analysis. First, the LiST model focuses only on mortality. Many of the add-on interventions studied also reduce morbidity, and some potentially important interventions, such as antihelminthic drugs, were not considered as their direct impact is exclusively on morbidity.^2^ Second, limited availability of data forced us to exclude two states of less than 2 million inhabitants each.^3^^,^^8^ Third, uncertainty concerning parameter values for vitamin A effectiveness, proportional mortality due to malaria, and compliance were found to influence mortality projections.^4^ Fourth, our mortality projections represent a specific point in time, whereas child survival and health services coverage are changing rapidly in India.^5^ Finally, due to constraints of logistics, it may not be possible in practice to offer as many add-on interventions as we have modelled for this analysis. Our primary purpose was to demonstrate the potential benefits of bundling proven interventions with a vaccination campaign. We also showed the utility of an evidence-based approach for planning add-ons for supplementary immunization activities. The integrated package studied in this analysis should be seen as aspirational. In practice, the impact on mortality will depend on the actual interventions offered and may be less than illustrated here.

Mass measles vaccination campaigns in many countries have offered additional interventions,^2^ but the choice of which interventions to include has generally been made in an ad hoc way rather than through a systematic analysis such as the one illustrated here. Although the interventions we examined were all deemed by Indian health planners to be technically feasible to incorporate into mass vaccination campaigns, implementation research is needed to assess the feasibility and impact on health systems of offering these interventions. We also need to assess the cost–effectiveness of supplementary immunization activities that include measles vaccine and add-on maternal and child health interventions in India. As vaccination campaigns must be repeated periodically, this research has the potential to revitalize political support for accelerated measles control strategies, as well as for other vaccines delivered through mass campaigns, such as rubella and polio.
